# Coping With Depressive Symptoms in Young Adults: Perceived Social Support Protects Against Depressive Symptoms Only Under Moderate Levels of Stress

**DOI:** 10.3389/fpsyg.2018.02780

**Published:** 2019-01-14

**Authors:** Myria Ioannou, Angelos P. Kassianos, Maria Symeou

**Affiliations:** ^1^Department of Psychology, University of Cyprus, Nicosia, Cyprus; ^2^Department of Applied Health Research, University College London, London, United Kingdom

**Keywords:** perceived social support, depressive symptoms, self-esteem, perceived stress, mediation, young adults

## Abstract

**Introduction:** The interrelationship between social support, depressive symptoms, stress and self-esteem in young adults remains unclear. This study aims to test the mediating role of self-esteem in the relationship between social support and depressive symptoms and the moderating role of perceived stress in the relationship between the two. This is important to inform components of future intervention development targeting youth depression.

**Methods:** Three hundred forty-four (*N* = 344) young adults in Cyprus aged 17–26 (78% female) completed measures of self-esteem, social support, depressive symptoms, and perceived stress. Structural equation models were used to examine the interactions between social support and depressive psychopathology, whereas mediational analyses were run to examine the mediating role of self-esteem. Latent moderated mediation models were applied to examine the potentially moderating role of perceived stress.

**Results:** Perceived social support from family and friends were significantly related to lower depressive symptoms. Self-esteem fully mediated the relationship between perceived family support and depressive symptoms. Perceived stress moderated the model, and perceived social support was found to be more protective against depressive symptoms when moderate levels of stress were presented.

**Conclusion:** The study demonstrates that social support is protective against depressive symptoms. Self-esteem and perceived stress are important mechanisms that interact with this effect. Implications include the efforts to increase perceived family support during college years and management of stress levels before working with depressive symptoms.

## Introduction

Perceived social support refers to how individuals perceive friends, family members and others as sources available to provide material, psychological and overall support during times of need. Perceived social support has been consistently related to well-being, as the perceived levels of support, love, and care can provide positive experiences (e.g., Siedlecki et al., 2014). A review suggested that high perceived social support is related to better physical and mental health outcomes as well (Uchino et al., 2013).

### Social Support and Relationships With Stress and Depressive Symptoms

Perceived social support and connectedness have been found to be stronger predictors of decreased depression in young adults than gender, self-esteem, and sleep quality (Armstrong and Oomen-Early, 2009). Numerous studies have been concerned with the role of perceived social support from parents, peers and the school in the reduction of depressive symptomatology in children and adolescents (e.g., Rawana, 2013).

Additionally, theoretical efforts have been made as well to understand the relationship between social support and depression. Based on the stress-mobilizing hypothesis, stress encourages individuals to seek social support (Singh and Dubey, 2015). However, one should note the high correlation between stress and psychological distress and especially the high comorbidity between stress and depression. This high comorbidity may explain a spurious positive relationship between depression and perceived social support (Starr et al., 2014).

The psychological pathways that mediate the association between perceived social support and mental health outcomes need to be further investigated (Uchino et al., 2013). This evidence is necessary in the effort to theoretically understand the functions of perceived social support, and to inform appropriate areas for interventions that could limit the negative effects of low social support on mental health. One important parameter involved in the relationship between social support and mental health is self-esteem.

### Perceived Social Support and Self-Esteem

According to the social-cognitive perspective, perceived social support promotes self-esteem, which subsequently leads to positive mental health outcomes (Lakey and Cohen, 2000). Perceived social support is suggested to be associated with positive thoughts about self; hence the direct and indirect impact on mental health outcomes through self-esteem. However, the way young adults think about social ties and support may activate different self-evaluations. For example, some may interpret social support as an indicator of their social acceptance and may activate positive self-schemas (e.g., a lovable person). On the other hand, others may interpret social support as an indicator of negative qualities (e.g., a weak person). Social support may trigger conflict and comparison with others, in cases that negative self-evaluations are produced after receiving social support. For instance, evidence suggests that perceived social support may actually carry a self-esteem threatening message at times, as receiving high social support may be interpreted as a sign of low coping ability, which in turn might increase distress (Choenarom et al., 2005). A more recent theoretical perspective suggests that providers of social support help the recipient through the regulation of affect, thought and action and that people who produce favorable affect and higher self-esteem to the recipients of their support are more likely to be perceived as supportive (Lakey and Rhodes, 2015). It is not clear under which circumstances social support could either enhance or threat self-esteem.

A bidirectional relationship between social support and self-esteem has been previously reported. Lee et al. (2014) found that social support mediates the relationship between self-esteem and depression, and at the same time self-esteem mediates the relationship between social support and depression. Perceived social support may improve psychological health through its effect on self-worth, sense of security and belonging, which are components of higher self-esteem. Social support provides a reassurance of self-worth, as it gives the perception that one is valued and accepted by others. At the same time however, the relationship between social support and self-esteem might be bidirectional. As an example, having low self-esteem may impact perceptions regarding social support and limit efforts to reach out for support, or may promote remembering negative aspects of past social interactions (Swann et al., 2003).

Kleiman and Riskind (2012) tested the mediating role of self-esteem in the relationship between perceived and reenacted social support, and suicidal ideation. They found that perceived social support was associated with increased self-esteem, and self-esteem increased the utilization of social support sources. The resulted model with reciprocal relations between self-esteem and social support partially buffered suicidal ideation. Recently, a longitudinal 4-year study with five waves in Australian adolescents (Marshall et al., 2014) showed that self-esteem was a stronger predictor of perceived social support quality and of the size of the social support network of the adolescents, compared to the reversed effect from perceived social support on self-esteem. This finding, however, may reflect a developmental effect, as adolescents place particular importance on positive self-beliefs that increase their confidence in relationships and results in the desire of more intimate relationships; hence the higher perceived social support quality.

### Summary of Previous Findings and Conflicting Evidence

Even though a number of studies suggested different mechanisms of the impact of perceived social support on mental health outcomes, the specific pathways through which perceived social support promotes young adults’ mental health are not well-known (Feeney and Collins, 2014). At the same time, some findings suggest that high perceived social support may not always promote mental health, but may actually be associated with increased distress (Seidman et al., 2006). Specifically, receiving high levels of support may activate self-doubting and low self-worth due to the perception that one is not capable to take care of himself (e.g., Lepore et al., 2008). Perceived social support may actually be a “mixed blessing” that helps to reduce negative outcomes for some people, but increases psychological distress for others (Bolger, 2014). Specifically, receiving more or less support that a person has provided (over-benefit and under-benefit, respectively) can be psychologically distressing, as this may be related to lower self-esteem and depressed mood.

Specific mental health problems may be related to lower perceived social support leading to conflicting evidence concerning the moderating role of stress in the relationship between social support and mental health outcomes. To illustrate, the perception of the stressful situation that one could not handle by themselves may limit the sense of mastery, productivity and functionality, leading to increased distress. Individuals with anxiety disorders may perceive that social support cannot buffer the negative impact of stress on mental health or protect against the impairments associated with anxiety disorders (Panayiotou and Karekla, 2013). Therefore, perceived social support may not be able to limit the effects of anxiety disorder on other mental health outcomes, such as depression. However, the interactions between social support, stress and depression are not always conclusive. For example, reviews of the literature point out an association between low perceived social support and poor mental health, which exists even if stress is not present (Lakey and Orehek, 2011), and not necessarily when there is comorbid stress (Lakey and Cronin, 2008).

Previous evidence suggests that only certain sources of support may have predictive utility after the experience of negative life events (Burton et al., 2004). Within this context, the examination of multiple sources of social support is important, though this has not been clearly emphasized in the literature. Also, the research exploring the importance of self-esteem in understanding the impact of perceived social support on mental health outcomes and particularly depression is still vague. Though a number of studies are concerned with the role of self-esteem in depression (for meta-analytic evidence see Sowislo and Orth, 2013), concurrent investigations controlling for perceived social support and perceived stress and the effect of self-esteem on depression and anxiety are limited.

### The Present Study

This study combines the evidence investigating mechanisms explaining the effect of perceived social support on depression, and the literature exploring the effects of stress levels on the relationship between depression and perceived social support. Previous studies suggest that clinical and subclinical depression affects a considerable population of young adults, i.e., university and college students (Armstrong and Oomen-Early, 2009; Lee et al., 2014). Also, during the transition to university or college, a number of major changes occur regarding the renewal and reorganization of students’ social network, which involves meeting new people and forming new intimate relationships. Perceived social support from family is also changed during this period of time, depending on student’s distance from home, previous relationships with parents and siblings and financial dependency. Therefore, we aimed to recruit young adults, in order to explore the interrelations between social support, self-esteem and depression. Given the diverse findings on the role of perceived stress, we aimed to explore how it can alter the interrelations between depression, social support and self-esteem.

We hypothesized that:

(a) Perceived social support would be related to lower levels of depressive symptoms.^1^
(b) Self-esteem would be a mediator of the relationship between perceived social support and depression.
(c) Perceived stress would be a moderator of the model between perceived social support, self-esteem, and depression. We expected that perceived social support would have a depression-buffering effect in cases of lower perceived stress (low and moderate levels of stress), since individuals with lower stress would be better able to keep people close to them and continue receiving support. As people with lower stress may perceive support as more positive than people with high perceived stress, we expected that perceived social support would be related to less distress for those young adults.

## Materials and Methods

### Participants and Procedure

This is an observational non-interventional study with low risk for participants. The Cyprus Bioethics Committee pre-screened and provided approval to the study. Participants were undergraduate and post-graduate students at the University of Cyprus in 2015, who provided informed consent to participate in a larger study involving the validation of the Multidimensional Scale of Perceived Social Support (MSPSS) in Greek (Zimet et al., 1990). The required sample size was computed through the G^∗^power software with the parameters of multiple regression (as we considered the existence of three predictors including perceived social support, perceived stress, and self-esteem). In order to be able to detect a small effect size of f^2^ = 0.05 the required sample was 312 participants [λ = 15.60, critical *F*(308) = 3.03, actual power = 0.95]. The participants were randomly selected from different university courses offered. We randomly selected courses from the weekly schedule and questionnaires were administered to these courses after the written consent of the courses’ directors and the verbal consent of the students. The average time for the completion of questionnaires was 15 min. The calculation of the intra-class correlation showed that ICC = 0.08 (95% CI 0.074, 0.093), supporting that individuals from the same group (i.e., class) were not similar in terms of their values and that multilevel management of the data (i.e., class, individual) was not advisable.

### Measures

The participants completed socio-demographic information, such as their age, gender, family status, living status, level and year of degree, and a battery of questionnaires.

#### Multidimensional Scale of Perceived Social Support

The MSPSS is a self-report 12-item instrument capturing the multidimensionality of perceived social support, through items that measure social support from family, friends and a special person (significant other). The three subscales of family, friend, and significant other perceived social support consist of four items, rated on a seven-point Likert scale ranging from “very strongly disagree” to “very strongly agree.” The subscales’ discriminant validity is satisfactory and the instrument has good psychometric properties in terms of validity and reliability index for all three subscales ranging from 0.85 to -0.92 and 0.87 to 0.93 for the whole scale (Budge et al., 2013). Sample items of the instrument include: “There is a special person with whom I can share my joys and sorrows” (support from a special-person/significant other subscale), “I can talk about my problems with my family” (family support subscale) and “I can count on my friends when things go wrong” (friends’ support subscale). The total score of the three subscales is summed to create the total score of perceived social support with higher scores indicative of higher perceived social support. The scale was translated to Greek using the forward-backward translation. It was forward translated from English to Greek by two authors (APK and MS) and back-translated to English by a third author (MI) who was not aware of the original items in English. Any discrepancies were resolved by consensus. Then, the scale was piloted with 10 students, who tested the clarity of the concepts and items of the scale.

#### Center for Epidemiological Studies – Depression Scale

The Center for Epidemiological Studies – Depression Scale (CES-D scale) (Radloff, 1977) is a self-report instrument consisting of 20 items measuring depression that cover affective, somatic, and cognitive and psychological symptoms. Responses are based on frequency of symptoms during the last week, on a four-point Likert scale ranging from “rarely or none of the time last week (less than 1 day of the week)” to “most or all of the time during last week (from 5 to 7 days of the week).” The items of the scale include “I did not feel like eating; my appetite was poor” (somatic symptom), “I had crying spells” (affective symptom), “I thought that my life had been a failure” (cognitive symptom) and “I felt depressed” (psychological symptom). The scale also includes four positively worded items, which were reversed and the scores in each item are summed up in order to form the total depression score, in which higher scores indicate higher depression. The CES-D scales’ reliability index ranges from 0.90 to 0.96. The Greek version has good psychometric properties including high internal consistency, high test–retest reliability and high sensitivity and specificity at the cutoff level of 23/24 for depressive disorder (Fountoulakis et al., 2001). Due to the present study focusing on a college population, depressive symptoms were treated as a continuous instead of a dichotomous outcome that would categorize individuals based on the depressive disorder cutoff.

#### Rosenberg Self-Esteem Scale

The Rosenberg Self-Esteem Scale (RSES) (Rosenberg, 1965) is a self-report instrument consisting of 10 items measuring self-esteem. The items are rated on a four-point Likert scale ranging from “strongly agree” to “strongly disagree.” The scale was validated using a Greek-speaking population in Cyprus (Panayiotou and Papageorgiou, 2007). Items include items such as “I feel that I’m a person of worth.” The negatively worded items are reverse scored and the scores in each item are summed up in order to form the total self-esteem score, in which higher scores indicate higher self-esteem. The scale has generally high reliability index ranging from 0.82 to 0.93.

#### Perceived Stress Scale-14

The Perceived Stress Scale-14 (PSS-14) (Cohen et al., 1983) is a self-report instrument consisting of 14 items assessing the perception of stressful experiences by asking individuals to rate how frequently they have feelings and thoughts related to events and situations that occurred during the previous month. The items are rated in a five-point Likert scale ranging from “never” to “very often.” The scale includes items such as “In the last month how often have you felt that you could not cope with all the things that you had to do?” The positively worded items are reversed and the scores in each item are summed up in order to form the total perceived stress score, in which higher scores indicate higher perceived stress. The scale has good psychometric properties with adequate internal consistency ranging from 0.77 to 0.90 (Lee, 2012). The PSS was validated in Greek in non-clinical settings (Andreou et al., 2011).

### Data Analysis

Data were screened for missing values and a missing value analysis was run to examine if there were systematically missing values in particular items. Correlation analyses between the variables under study were conducted. Due to the potential conceptual overlap between depression and self-esteem – as self-esteem is considered an identifying symptom of depression-, the correlations were calculated both using the total depressive symptoms and the depressive symptoms excluding the ones overlapping with self-esteem measure (questions 4 and 9 of the CES-D). Related to the above, partial correlations were calculated for among the variables due to the high comorbidity between anxiety, depression and self-esteem, as observed in previous studies as well (e.g., Hranov, 2007). Both exploratory factor analyses and confirmatory factor analyses (CFAs) were run in order to examine and confirm the factorial structure of the questionnaires. Then, structural equation modeling (SEM) and multi-group analyses were used to test the hypotheses of the present study. SEM was preferred against mediate regression models, in order to concurrently examine mediating and moderating variables while concretely accounting for measurement error and to avoid Type I error (Byrne, 2009).

To test if self-esteem mediated the relationship between social support and depression we evaluated if the direct effect (from social support to depression) remained significant after adding self-esteem in the model and also evaluated the change in the variance of depression explained. Two ways are proposed for the examination of the moderating role of perceived stress, when using Mplus. Specifically, when latent variables are involved, the examination of the interaction between the moderating variable and the mediational variable is suggested. Latent moderated structural equations (LMS) have been showed to be accurate in terms of estimated effects and confidence intervals, when compared to regression, which underestimates the magnitude of effects and provides inaccurate confidence intervals (Cheung and Lau, 2015). At the same time, multi-group analyses can be performed as well, which correspond in type to Hayes’ PROCESS analysis (Stride et al., 2015), with the aim to examine the changes in the model for those with low, moderate and high levels of the moderating variable (i.e., perceived stress in this case). The way that the three groups are formed is based on the mean score of the variable and the standard deviation (i.e., one SD above and below the mean represent the moderate level, one SD below constitutes the low level and one SD above is considered the high level). Both ways to approach latent moderated mediation testing were applied for the aims of the study.

Firstly, we reversed the items that were positively worded in order to be able to sum the items of the PSS, in a way that higher scores indicate higher perceived stress. The maximum score that any participant could get was 70 and the minimum was 14. The cutoff scores for the aims of the study were 36, 41.375 and 46.75, respectively for low, moderate and high perceived stress.

Model fit was *a priori* decided to be evaluated with the chi-square test, as well as the following approximate fit indices: the Root Mean Square Error of Approximation (RMSEA), the parsimony corrected (PCLOSE), the Bentler’s Comparative Fit Index (CFI), the Tucker Lewis Index (TLI), and the Standardized Root Mean Square Residual (SRMR). For an adequate model fit, most of the indices should be met, with the CFI > 0.95, the PCLOSE close to 1, the TLI > 0.95, and the RMSEA and SRMR < 0.05, with < 0.08 considered satisfactory as well (Hu and Bentler, 1999). Bayesian Information Criterion (BIC) was examined (estimated using maximum likelihood ML estimator) as an index of parsimony (difference > 10 was considered very strong evidence that the model with the lower BIC value was better than the comparison models) (Raftery, 1995). All data analyses were conducted using Mplus version 7.3 (Muthén and Muthén, 1998–2012), using the weighted least square mean and variance adjusted (WLSMV) which is a robust estimator for ordinal data that do not assume variables with normal distribution (Brown, 2006). We exploited all available data including the ones partially missing (<90% of the items missing), under the assumption of data missing at random (Little and Rubin, 2002).

## Results

### Demographic Information

Three hundred and fifty participants completed the questionnaires. Two participants who had missing values in more than 90% of the items were removed, and four participants which were outliers were not included in the subsequent analyses. The resulting sample consisted of 344 (*N* = 344) participants. The age of the participants ranged from 17 to 26, with *M* = 20.78 (*SD* = 3.94). The 94% of the sample were undergraduate students, and 78% were female, which approximates the percentage of female college students in Cyprus (Meletiou-Mavrotheris and Maouri, 2011). A percentage of 7% of the sample were married and 6% had children. Correlations between the variables under study were in the expected direction (Table 1).

**Table 1:** Correlations between the total scores of the variables under study.

	2	3	4	5	6	7	8
(1) MSPSS_Family	0.405***	0.451***	0.798***	-0.307***	-0.315***	-0.326***	-0.184**
(2) MSPSS_Friend	-	0.429***	0.791***	-0.250***	-0.146**	-0.334***	-0.310***
(3) MSPSS_Significant other		-	0.769***	-0.142**	-0.145**	-0.262***	-0.224***
(4) MSPSS_Total			-	-0.261***	-0.303***	-0.394***	-0.309***
(5) PSS-13 perceived stress				-	0.619***	0.776***	0.631***
(6) RSES self-esteem					-	0.607***	0.585***
(7) CES-D depressive symptoms						-	0.990***
(8) Modified CES-D (excluded items overlapping with SE and partial correlations provided when controlling for SE)							

*^∗∗^*p* < 0.01, ^∗∗∗^*p* < 0.001.*

### Examination of the Factorial Structure of the Questionnaires

The exploratory factor analyses of the scales showed the expected factors and provided the required support to proceed with the CFAs. For the CFAs of the scales, a multi-trait multi-method (MTMM) procedure was used, by considering the method factors in case the scales included positively and negatively worded items. The CFA analyses confirmed the questionnaire’s structure and are available as Supplemental Online Material.

### Associations Between Perceived Social Support, Self-Esteem, and Depression

**FIGURE 1:**
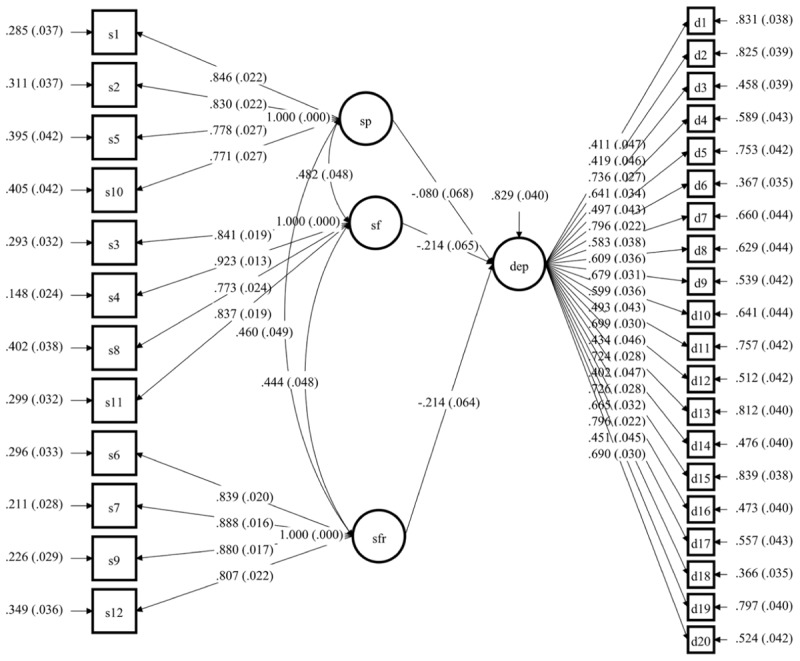
Model A which shows the effect of perceived social support on depressive symptoms. All the estimates presented are the standardized estimates. Latent factors: dep- depressive symptoms; sp- perceived social support from significant person; sf- perceived social support from family, sfr- perceived social support from friends.

A model with effects from the three sources of social support on depressive symptoms with the sample of 344 participants was tested (Model A), showing a good fit, with χ^2^(447) = 707.588, *p* < 0.001, CFI = 0.956, TLI = 0.952, RMSEA = 0.041 (90% CI 0.035, 0.047), SRMR = 0.047, BIC = 24862.483. The model A showed that perceived social support from family (*b* = -0.214, *p* = 0.002) and friends (*b* = -0.214, *p* = 0.002) was significantly negatively associated with depressive symptoms (Figure 1). Perceiving social support from a significant other had a non-significant effect on depression (*b* = -0.080, *p* = 0.238). Having higher support from family and friends was related to lower feelings of depression. Perceived social support accounted for 17% of the variance of depression (*R*^2^ = 0.171, *p* < 0.001). The model with self-esteem as a mediator of the impact of social support on depression had a good fit (Model B), with χ^2^(796) = 1162.783, *p* < 0.001, CFI = 0.946, TLI = 0.940, RMSEA = 0.045 (90% CI 0.041, 0.050), SRMR = 0.056, BIC = 31390.879. Model B showed that when taking self-esteem into account, perceived social support from family stopped having a significant effect on depression (*b* = -0.044, *p* = 0.470). Family support had a significant positive effect on self-esteem (*b* = 0.311, *p* < 0.001) and self-esteem had a significant negative effect on depression (*b* = -0.550, *p* < 0.001). The effect of social support from friends on depression was reduced but remained significant (*b* = -0.223, *p* < 0.001) and perceived friend support did not have an impact on self-esteem (*b* = 0.010, *p* = 0.888), showing that self-esteem was not a significant mediator of the relationship between friend support and depressive symptoms. Based on the model, having high family support is related to feeling less depressed, partly because it increases your sense of self-esteem. The consideration of self-esteem in the model added on the variability of depression explained, as squared multiple correlations showed that from the 17% of the variance of depression explained by model A, the variance explained by model B increased to 55% (*R*^2^ = 0.547, *p* < 0.001). The variance of self-esteem that could be explained by perceived social support was 10% (*R*^2^ = 0.101, *p* < 0.001). The findings were retained even after controlling for age and gender. After this consideration, the model explained 57% of the variance of depression (*R*^2^ = 0.572, *p* < 0.001) and 49% of the variance of self-esteem (*R*^2^ = 0.492, *p* < 0.001). Being older (*b* = 0.179, *p* < 0.001) and female (*b* = -0.125, *p* = 0.050) was related to higher depression. Relatively, being younger in age (*b* = -0.100, *p* = 0.027) and male (*b* = 0.616, *p* < 0.001) was related to higher self-esteem. Being male was related to having higher perceptions of social support from family (*b* = 0.235, *p* < 0.001), friends (*b* = 0.138, *p* < 0.001) and a significant other (*b* = 0.144, *p* = 0.017). Age marginally differentiated only the levels of social support from family, as being older in age was related to lower family support (*b* = 0.113, *p* = 0.046).

### Examination of the Moderating Role of Perceived Stress

The examination of the moderating role of perceived stress, was firstly applied using the interaction terms between the mediating variable (i.e., latent factor of self-esteem) and the moderating variable (i.e., latent factor of perceived stress), after controlling for the effect of the types of perceived social support on depressive symptoms. The interaction term had a significant effect on depressive symptoms (*b* = -0.689, *p* < 0.001) and the log-likelihood change was significant (LL H0 = -14885.173, *p* < 0.001, BIC = 30687.327). Further examination of the multi-group latent moderated mediation model was conducted, in order to investigate the type of interaction found between self-esteem and perceived stress.

The findings supported that the model reflecting the mediational effect of self-esteem in the relationship between perceived social support and depressive symptoms, was not invariant across all groups (i.e., low, moderate, and high levels of perceived stress). The log-likelihood change was significant (LL H0 = -21329.892, *p* < 0.001, BIC = 43795.710). Even though the interaction between self-esteem and perceived stress had a significant effect on depressive symptoms (*b* = -0.595, *p* = 0.004), the interaction between perceived social support and perceived stress did not have a similar significant effect (*b* = -0.003, *p* = 0.852). That is, the direct protective effects of perceived social support on depressive symptoms were not significantly moderated by perceived stress. However, the indirect effects of support on depressive symptoms through self-esteem were differentiated based on the levels of perceived stress. The confidence intervals of the effects on depressive symptoms are presented on Table 2.

**Table 2:** Confidence intervals of the model results.

	Lower 0.5%	Lower 2.5%	Lower 5%	Estimate	Upper 5%	Upper 2.5%	Upper 0.5%
Self-esteem	-1.686	-1.469	-1.357	-0.777	-0.197	-0.115	-0.065
SEXPSS	-1.133	-1.005	-0.939	-0.595	-0.251	-0.185	-0.057
Social support	-0.126	-0.109	-0.101	-0.086	-0.056	-0.011	-0.003
Perceived stress	0.179	0.206	0.220	0.291	0.362	0.375	0.402
SSXPSS	-0.049	-0.038	-0.033	-0.003	0.026	0.032	0.043
Age	-0.072	-0.064	-0.060	-0.040	-0.019	-0.015	-0.007
Gender	-0.055	-0.044	-0.038	-0.008	0.023	0.029	0.040

*Only the effects on depressive symptoms are presented. SEXPSS, interaction term between self-esteem and perceived stress; SSXPSS, interaction term between perceived social support and perceived stress.*

**FIGURE 2:**
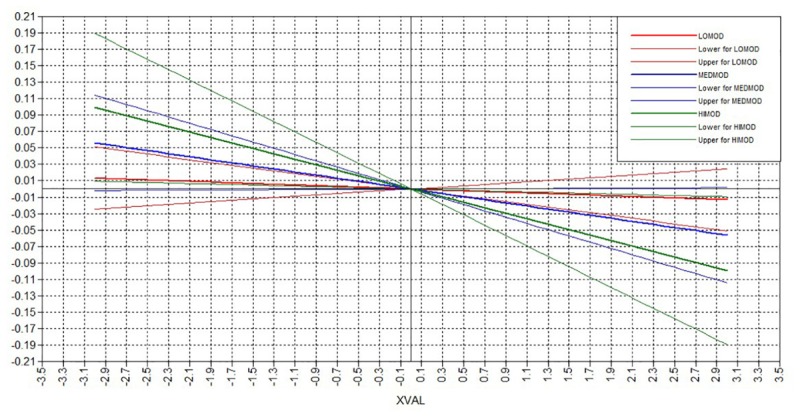
Plot of the indirect effects. Lines represent the low (red), moderate (blue), and high (green) levels of perceived stress, with their lower and upper confidence intervals.

Social support did not boost self-esteem which could subsequently decrease depressive symptoms to a similar extent for those with low, moderate, and high levels of perceived stress. The effect for those with low levels of stress traversed the zero axis, supporting that the indirect effects were not significant. The effect for those with high levels of perceived stress was significant and the slope was larger, suggesting low impact of perceived social support on self-esteem and low decrease of depressive symptoms under high stress. Doubled levels of perceived social support were needed to produce an increase in self-esteem and a subsequent decrease in depressive symptoms for those with high perceived stress, compared to those with moderate perceived stress. On the other hand, those with moderate levels of perceived stress could experience a rather stable significant effect of perceived social support on self-esteem and of self-esteem on depressive symptoms. The slopes of the indirect effects based on the moderating effects of perceived stress are presented on Figure 2.

## Discussion

The present study examined self-esteem and perceived stress, as factors explaining, and interacting, respectively, with the effects of perceived social support on depression. Separate examination of the direct effects of different sources of perceived social support, showed that only the social support received from family and friends and not the perceived support from a significant other were negatively related to depressive symptoms. Family and peer support seemed to impact depression through different pathways, as self-esteem was found to be a mediator of the effect of family support on depressive symptoms, but the same did not stand for peer support. This finding may indicate the differential impact of perceived social support on personal characteristics and might be developmentally sensitive. For example, perceiving social support from friends may not impact on a young adult’s self-esteem, as friends’ support may be considered more stable, important and granted at this developmental stage (Walen and Lachman, 2000). In the same line, since the relationships with parents and family are subjected to change and adaptation during college years, continuation or re-establishment of family support might be very powerful during these years, as support from family may not be assumed for the general population of young adults.

There is support for the depression-buffering hypothesis, though the indirect effect of social support on depression through self-esteem is subjected to and moderated by the levels of perceived stress. Our findings suggested that those with high levels of perceived stress perceive very low social support that has subsequently lower benefits on increasing their self-esteem and decreasing their depressive symptoms. This is in line with the finding that perceived social support does not decrease depression in people with anxiety disorders (Panayiotou and Karekla, 2013). Based on the indirect effect regression slopes, very high levels of perceived social support are needed for people with high levels of stress in order to be able to achieve the protective effects of support on self-esteem and depressive symptoms. People with high levels of stress may be less able to keep contact and tight social connections with other people; thus, keeping others in distance probably because they convey their high stress to their social networks (Coyne and Downey, 1991). The absence of social connections when stress levels are elevated further prevents people from the opportunity to achieve meaning making that could activate them to deal with depressive symptoms (Dulaney et al., 2018). In this framework and having in mind the difference between perceived and actual social support, it is also possible that people with high levels of perceived stress underestimate the social support they receive, and/or are not able to use support in a way that could maximize the benefits for their self-esteem and psychological well-being. It could also be that the increase in their self-esteem is more context-specific when achieved, and not easily transferred and generalized to lower depressive symptoms due to cognitive limitations or errors related to their high levels of stress (e.g., selective attention, intolerance of uncertainty, worry, rumination and catastrophizing, external rather than internal attribution of support and of increased self-esteem).

On the other hand, the effects of perceived social support on self-esteem and depressive symptoms are not significant for those with low perceived stress. That is, people with low stress, may seek less social support, as they might feel more self-competent regardless of the extent of perceived social support. Due to the high comorbidity between depression and anxiety (Sowislo and Orth, 2013) individuals with low levels of stress may also have lower depression levels, and may not seek social relationships in order to improve their mental health as they feel able to take care of themselves (Lepore et al., 2008). These individuals may be more independent and autonomous.

People with moderate levels of perceived stress were able to benefit the most from the effects of social support on self-esteem and the increases of self-esteem further lowered their depressive symptoms. Keeping moderate levels of perceived stress may result in effecting self-monitoring to reach out for social support when needed and to recognize the effects of this support for psychological well-being (in terms of self-esteem and depressive symptoms). At the same time, having moderate levels of stress may be related to more objective estimations of the benefits of perceived social support, compared to when having low or high levels of stress (in both of which underestimation or distorted attribution of the need or the benefits of social support is more plausible).

Overall, this study suggests that self-esteem is important in trying to understand the relationship between perceived social support and depression. Self-esteem significantly mediates the relationship between family support and depression, and also moderates the relationship between perceived support from a special person and depression. However, the level of perceived stress is an important parameter that can completely shape the effects of social support on self-esteem and especially the effects of self-esteem on depressive symptoms. This seems to be also in line with clinical work suggesting limited effect of, or need for longer, treatment of depression when there are comorbid anxiety disorders, regardless of the existence of a social support network (Doss and Weisz, 2006).

Even though the significant direct effects of perceived social support on depression were retained, the moderated mediation found suggests the complex effects of perceived stress on the mechanisms interplaying between social support and depressive symptoms. Consideration of the moderating effects of stress on the interplaying mechanisms is very important, especially when having in mind the significant increase offered by self-esteem in the present study in regard to the explained variance of depressive symptoms.

### Limitations

One limitation of our study is the over-representation of female participants. This is a common problem in studies using college population. However, gender was inserted in the models as a covariate, in order to account for the effects of the over-representation of females. Another limitation is that other potentially moderating factors were not measured and should be explored in the future, such as socio-economic status, physical illness, and history of mental health problems. The findings of this study might be culture-specific, as family bonds are considered considerably strong in this population (e.g., Georgiou, 1996). The cross-sectional design of the study limits the conclusions that could be drawn regarding the bi-directionality of the effects between perceived social support and depression. However, the direction of effects was based on the findings of a pilot study on a college population, as it was found that the reversed pathway from depression to perceived social support produced standardized estimates of lower significance and magnitude. Also, this is the first study using the stress-buffering hypothesis to assess how stress relates to social support and depression and suggests that a longitudinal study investigating this further is feasible and necessary. Importantly, one should note that the population was non-clinical. Due to the use of a college sample, one should be cautious with the interpretation of the findings as evidence for depressive symptoms, instead of evidence for clinically-diagnosed depression. More research is needed to investigate if the protective effects of perceived social support could be generalized to clinical populations struggling with depression. The use of MSPSS which is scored on a seven-point scale potentially increases the risk for extreme response style (ERS). However, not enough support for ERS was provided based on the data, as the scale had good psychometric properties, the correlations between the items were in the appropriate range, and the proportion of individuals with consistent extreme responses was low (<25%). Lastly, the moderated mediation using the multi-group approach for low, moderate, and high stress was applied using the latent factor for social support from all three sources of family, friends, and significant person. Due to the complexity of the moderated mediation models, concurrent examination of the effects using all three latent social support sources is not advisable (e.g., Muthén and Muthén, 1998–2012).

## Conclusion

The present study adds to the literature investigating the interrelations between perceived social support, perceived stress, self-esteem, and depression. We employed latent structural equation modeling to concurrently test mediating and moderating effects while accounting for the measurement error. With this analysis, we avoided type I error implicated in previous studies that had tested various models to separately test the effects of different social support sources on different mental health outcomes. The use of a multidimensional measure of perceived social support adds to the strengths of the study, as it allowed the distinct investigation of the role of each perceived social support source.

Clinical implications of this study are relevant to the development of interventions for mild to moderate depressive symptoms during college years that involve a considerable degree of family communication and support. Practical suggestions to families during college years should not be discarded, as family support during college seems to enhance self-esteem and potentially decrease any negative self-evaluations they have and subsequently depressive symptoms. At the prevention level, our findings show the need to educate families to remain close to their offspring even when they are in the developmental stage of early adulthood and are becoming more autonomous, and to keep using parental practices that may increase self-esteem, such as quality communication showing interest for their lives away from home and trust for their personal choices, emotional availability under periods of stress, and positive reinforcement. Theories of popularity-socialization may help further understand this transition and the role of support in coping with depressive symptoms (Reynolds and Crea, 2015).

Important implications stem from the findings of the moderating effect of perceived stress, and its interactions with self-esteem and perceived social support. Stress management and practice on relaxation techniques may need to be applied first in cases with comorbid depressive and anxious symptomatology, before dealing with depression symptoms, low self-esteem, and/or negative self- and others- evaluations. Even though social support may be beneficial even when under high levels of stress, double levels of perceived support are needed to achieve its protective effects, compared to when having moderate levels of stress. Therefore, working with stress management before depressive symptoms may provide skills (e.g., realistic estimation of risk, de-catastrophizing) that will enhance the empowering effects of social support on self-esteem and subsequently on lowering depressive symptoms. After effective stress management, youths can become more functional and motivated to work with their depressive symptoms.

Future longitudinal studies investigating other mechanisms apart from self-esteem through which perceived social support can impact depressive symptoms, need to consider the potentially moderating role of perceived stress. Also, the examination of multiple sources of perceived social support is important. Implementation of similar latent moderated mediation models with clinical populations with depression is highly recommended for future investigations. The findings of the present study have provided important pilot evidence for non-clinically elevated depressive symptoms that could drive research toward this direction.

## Author Contributions

AK and MS conceived and designed the study. MS and MI coordinated the collection of data. MI analyzed the data and prepared the first manuscript. All authors provided critical comments and approved submission.

## Conflict of Interest Statement

The authors declare that the research was conducted in the absence of any commercial or financial relationships that could be construed as a potential conflict of interest. The reviewer JW and handling Editor declared their shared affiliation.

## Footnotes

1 The literature on the relationship between social support and depression is not conclusive regarding the direction of the relationship. However, a previous pilot study of our research team applied a cross-lagged longitudinal design and supported that the effect of perceived social support on depression was significant (*b* = -0.19, *p* = 0.002), and that the reversed reciprocal effect of depression on perceived social support was smaller in magnitude and non-significant (*b* = -0.03, *p* > 0.05). The direction of the relationship and the formation of the first hypothesis were based on the pilot data.
