# Supplementary material for: Coping With Depressive Symptoms in Young Adults: Perceived Social Support Protects Against Depressive Symptoms Only Under Moderate Levels of Stress
Source: Front Psychol. 2019 Jan 14;9:2780. doi: 10.3389/fpsyg.2018.02780 (PMC6340372; doi:10.3389/fpsyg.2018.02780)
Supplement: Supplementary file 1 [file Table_1.docx]

**Supplemental Online Material**

**The Confirmatory Factor Analyses of the included questionnaires**

The CFA of the CES-D scale included three latent factors, namely depression, and two method factors for negatively and positively worded items. The model had an excellent fit, with χ^2^ (151) = 240.138, p< .001, CFI= .936, TLI= .920, RMSEA= .059 (90% CI .044, .072), SRMR= .055, BIC= 7670.916. The model was significantly better than the model with one method factor for the positively worded items (Δχ^2^ (16) = 67.868, p< .001, ΔBIC= 68.571), and the model with no method factors (Δχ^2^ (19) = 113.468, p< .001, ΔBIC= 15.665). The CFA for the RSES scale included three latent factors as well, one for self-esteem and two method factors for positively worded and negatively worded items and had an excellent fit, with χ^2^ (23) = 36.855, p= .034, CFI= .985, TLI= .970, RMSEA= .042 (90% CI .012, .066), SRMR= .024, BIC= 6359.072. This model had significantly better fit than the model with only one method factor for positively worded items (Δχ^2^ (5)= 32.146, p< .001, ΔBIC= 12.528), and the model with no method factors (Δχ^2^ (9)= 33.04, p< .001, ΔBIC= 3.565). The CFA for PSS-14 was tested using again a latent factor for perceived stress and two method factors for negatively and positively worded items resulted in an excellent model fit, with χ^2^ (61) = 109.101, p= .002, CFI= .972, ΤLI= .958, RMSEA= .048 (90% CI .033, .062), SRMR= .041, BIC= 11458.672. This model had significantly better fit than the model with one method factor for positively worded items (Δχ^2^ (7) = 203.862, p< .001, ΔBIC= 162.977) and the model without method factors (Δχ^2^(13) = 213.385, p< .001, ΔBIC= 137.457). The CFA of the MSPSS had acceptable fit, with χ^2^ (51) = 153.592, CFI= .964, TLI= .953, RMSEA= .078 (90% CI .064, .092), SRMR= .032, ΒΙC= 10250.906. The correlations between the three first-order factors were moderate, ranging from r= .445 to r= .492 and the loadings of the first-order factors on the second-order factor of social support ranged from b = .438 to b = .497, showing limited support for a second-order CFA model of MSPSS.
